# Circadian Variation in Human Milk Hormones and Macronutrients

**DOI:** 10.3390/nu15173729

**Published:** 2023-08-25

**Authors:** Majed A. Suwaydi, Ching Tat Lai, Alethea Rea, Zoya Gridneva, Sharon L. Perrella, Mary E. Wlodek, Donna T. Geddes

**Affiliations:** 1 School of Molecular Sciences, The University of Western Australia, Crawley, WA 6009, Australiaching-tat.lai@uwa.edu.au (C.T.L.); zoya.gridneva@uwa.edu.au (Z.G.); sharon.perrella@uwa.edu.au (S.L.P.); m.wlodek@unimelb.edu.au (M.E.W.); 2 School of Applied Medical Sciences, Jazan University, Jazan 45142, Saudi Arabia; 3 Mathematics and Statistics, Harry Butler Institute, Murdoch University, Murdoch, WA 6150, Australia; alethea.rea@murdoch.edu.au; 4 Department of Obstetrics and Gynaecology, The University of Melbourne, Melbourne, VIC 3010, Australia

**Keywords:** human milk, lactation, breastfeeding, hormones, adipokine, macronutrient, circadian rhythm, diurnal rhythm

## Abstract

There is an inadequate understanding of the daily variations in hormones and macronutrients in human milk (HM), and sample collection protocols vary considerably from study to study. To investigate changes in these milk components across 24 h, 22 lactating women collected small milk samples before and after each breastfeed or expression from each breast. Test weighing was used to determine the volume of HM consumed in each feed. The concentrations of leptin, adiponectin, insulin, fat, and glucose were measured, and the intakes were calculated. A linear mixed model was fitted to assess within-feed and circadian variation in HM feed volume and concentration, and intakes of several components. The average infant intake of HM was 879 g/24 h. Significantly higher pre-feed concentrations were found for adiponectin and glucose and lower post-feed concentrations were found for insulin and fat. Significant circadian rhythms were displayed for leptin, adiponectin, insulin, glucose (both concentration and intake), fat concentration, and milk volume. These findings demonstrate the necessity for setting up standardised and rigorous sampling procedures that consider both within-feed and circadian variations in HM components to gain a more precise understanding of the impacts of these components on infant health, growth and development.

## 1. Introduction

Human milk (HM) is a complex biofluid that provides essential components for infant growth and development. This complexity supports the fundamental concept of HM as biological system that intersects and interacts with maternal factors and influences infant health and development [1,2,3,4,5,6,7,8]. Breastfeeding is recommended as the sole source of infant nutrition for the first 6 months of life, continuing up to 2 years or more [9,10]. HM composition is highly dynamic in relation to lactation stage, pre- and post-feed variations, and circadian variations [11,12,13]. There has been increased focus on the potential for many HM components to demonstrate a circadian rhythm [11,14]. These fluctuations may play a role in infant growth and the development of their biological clock in addition to having implications for HM sampling and analysis.

In general, the human body follows a 24 h circadian rhythm guided by the suprachiasmatic nuclei, which is located in the hypothalamus [15,16]. HM composition depicts circadian fluctuations that are likely to transfer time-related information from the mother to her newborn. This makes HM a unique chrono-nutrition factor [14] that may help newborns to communicate with their external environment.

Components such as hormones, fat, and glucose that are important for infant physiological processes may have within-feed and circadian concentration variations (rhythms) that may have implications on sampling of HM for research studies. Previous research indicated that several HM components, such as hormones, exhibit a circadian rhythm [11,17,18]. Therefore, any within-feed or circadian variations in the concentrations of these components may impact research outcomes and the interpretation or comparison of results between studies [13,19]. A considerable number of HM studies do not report the timing of sample collection in relation to feeding, such as pre-, mid-, or post-feed, or breast expression, and samples collected at unknown time points are generally used for analysis [19,20]. Sampling in relation to the time of day is rarely investigated, and several studies have provided weak to moderate evidence that leptin, fat, and glucose vary throughout a 24 h period [11]. Conversely, components such as carbohydrates and protein are relatively stable throughout a 24 h period [11]. Additionally, most studies that aimed to investigate 24 h variations in HM components did not use adequate methodology to assess a biological time series. Samples were usually collected at limited time points throughout the 24 h, sometimes with broad collection times reported such as morning, day, evening, and night. Further, the fully breastfed infant’s HM daily intake ranges from 478 to 1356 g/24 h [21], and the majority of the infants (64%) breastfeed at night and have larger volumes at night compared to morning, day and evening which could be explained by the reduced feeding frequency related to infant stomach capacity and/or gastric emptying rate. Furthermore, the breastfed infants’ gastric emptying and breastfeeding patterns are influenced not only by volumes but also by HM composition and the intake of components [22], suggesting possible effects of HM circadian variations. When investigating the impact of circadian variation in concentrations of HM components, infant milk intake should be measured in order to improve our understanding of HM as chrono-nutrition.

The lack of consideration of the timing of HM sample collection may somewhat explain the inconsistent results between studies. This study aimed to investigate within-feed differences and circadian variation (24 h) in the concentrations and intakes of the HM leptin, adiponectin, insulin, fat, and glucose.

## 2. Materials and Methods

### 2.1. Study Participants and Study Design

Healthy mothers (*n* = 22) of singleton term-born infants at 4 to 6 months postpartum were recruited for this study from the community and existing online networks. All mothers provided written informed consent for participation in the study, which was approved by the Human Research Ethics Committee, The University of Western Australia (RA/4/1/4492). Maternal and infant demographics were recorded, 24 h milk profiles were measured by mothers at home using digital scales, and pre-/post-feed and expression milk samples (*n* = 505) were collected. Infant 24 h intakes of HM leptin, adiponectin, insulin, fat, and glucose were calculated by averaging the pre- and post-feed concentrations and subsequently multiplying by the HM intake (volume) for the corresponding feed.

### 2.2. Measurement of 24 h Milk Intake and Sample Collection

The 24 h milk profiles were measured by mothers at their home using accurate digital scales (BabyWeigh™, Medela Inc., McHenry, IL, USA, resolution 2 g, accuracy ± 0.034%) [13]. Mothers used baby-weigh scales to weigh either their infant before and after each breastfeed or the collection bottle before and after each breast expression. During the same 24 h period, mothers hand-expressed or pumped small (1–2 mL) milk samples into 5 mL polypropylene plastic vials (Disposable Products, Adelaide, SA, Australia), promptly before and after each breastfeed and/or breast expression. The samples were labelled and immediately stored in the home freezer, and then transported on ice to the laboratory at The University of Western Australia and stored at −20 °C until analysis.

### 2.3. Biochemical Analysis of Human Milk

Milk samples were prepared for biochemical analysis (leptin, insulin, and adiponectin) by thawing at room temperature (22 °C) and then homogenized using beads homogenizer (BeadBug^TM^ 6 Position Homogenizer (Benchmark Scientific, Sayreville, NJ, USA) at 3 cycles of 5 s each. After homogenization, the samples were aliquoted into 0.5 mL polypropylene microcentrifuge tubes (SSIbio, Lodi, CA, USA) with the required volumes to measure the milk components of interest in duplicate.

Leptin concentration in whole milk samples was analyzed using Human Leptin ELISA DuoSet (DY398, Lot: P262874, R&D systems, Minneapolis, MN, USA). The kit was optimized to measure milk leptin using Greiner Bio-One 96-well half area, high binding ELISA plates (Item No.: 675061, Greiner Bio-One GmbH, Kremsmünster, Austria). Plates were prepared by pipetting 50 µL of capture antibody per well and incubated overnight at room temperature (22 °C). The plates were washed 3 times with washing buffer, dispensed at 200 μL per well, using a plate washer, then blocked by adding 150 µL of reagent diluent and incubated for a minimum 1 h. Next, the plates were washed 3 times, and 50 µL of standards (concentration range 0.9–0.014 ng/mL), samples, and internal quality control were added in duplicate and incubated for minimum 2 h at 25 °C. Then, the plates were washed 3 times and 50 µL of detection antibody was added and incubated for minimum 2 h at 25 °C. Following the second incubation, 50 µL of Streptavidin-HRP was added and incubated for a minimum of 20 min at 25 °C in a dark area. Next, the plates were washed, followed by the addition of 50 μL of substrate colour reagent, and incubated for 20 min at 25 °C in a dark area. The reaction was stopped using 25 µL sulphuric acid stop solution. Absorbance was read at 450 nm using spectrophotometer (Enspire Multimode Plate Reader, Waltham, MA, USA). The recovery average of leptin was 97.9 ± 6% (*n* = 5, coefficient of variation (CV) = 2.8%) with a detection limit of 0.014 ng/mL, and the average intra- and inter-assays CV were 1.62% and 10.01%, respectively.

Adiponectin concentration in whole milk samples was analyzed using the Human Adiponectin ELISA, High Sensitivity (RD191023100, Lot: E21-040, BioVendor, Brno, Czech Republic) following the protocol (b) that the manufacturer recommended for use to measure milk adiponectin concentration. The recovery average of adiponectin was 96.2 ± 3.2% (*n* = 10) with a detection limit of 0.156 ng/mL [22]. The average intra- and inter-assay CV were 6% and 11.97%, respectively.

Insulin concentration in whole milk samples was analyzed using the human Insulin ELISA BioVendor (RIS006R, Lot: X21-136S01, BioVendor, Brno, Czech Republic) following the manufacturer’s protocol. The recovery average of insulin was 100.33 ± 2.82% (*n* = 5, CV = 2.81%) with a detection limit of 0.17 µIU/mL. The average intra- and inter-assay CV were 2.34% and 15.8%, respectively.

Fat concentration (%) was measured using the creamatocrit method [23], and the cream percentage was converted to g/L using the following formula:

Fat (g/L) = 3.968 + (5.917 × Creamatocrit (%))(1)

After measurement of the creamatocrit percentage, the glass capillary was cut open to obtain the skim milk for glucose analysis. Glucose concentration in skim milk samples was analyzed using D-Glucose HK Assay Kit (K-GLUHK-220A, Lot:200218-8, Megazyme, Wicklow, Ireland). Briefly, the appropriate volume of reaction solution (210 µL per well) was prepared immediately before analysis based on the manufacturer’s protocol containing buffer, NADP^+^/ATP and distilled water. Skimmed milk samples (diluted in distilled water 1:2), standards and controls were pipetted (10 µL) into wells on a 96-well microtiter plates that contained 210 µL of prepared reaction solution. The plates were mixed for 3 min at 25 °C using a plate shaker (PST-60HL, BioSan, Riga, Latvia). Absorbance (A_1_) was measured at 340 nm on a plate spectrophotometer (Enspire Multimode Plate Reader, Waltham, MA, USA). Then, 2 µL of suspension 3 (Hexokinase and glucose-6-phosphate dehydrogenase) was pipetted into each well, mixed and incubated for 5 min. Absorbance (A_2_) was read at 2 min intervals until the reaction reached the plateau. Concentrations of glucose in skimmed milk samples were calculated using ΔA (A_2_ − A_1_) based on calibrated standard curve (range 720–11 µg/mL). The recovery of glucose was 100.2 ± 2% (*n* = 9), the glucose assay detection limit was 0.66 µg/mL. The average intra- and inter-assays CV were 5.9 and 6.77%, respectively. The results were converted into mmol/L using the molecular weight of glucose (180.16 g/mol).

### 2.4. Statistical Analysis

The data were analyzed and visualized using R Statistical Software 4.1.2. and presented as mean ± standard deviation (SD) unless specified. A linear mixed model was fitted to investigate the within-feed variation and circadian variation in milk feed volume, components concentration and intake with a circadian cycle (cosine and sine terms) for each mother as a random effect. The explanatory variables considered were a circadian cycle (cosine and sine terms) for concentration and intake outcomes, and an indicator variable for pre- or post-feed. Visualizations included the sample estimates of the population for both pre- and post-feed measures and circadian cycles for each mother post-feed only.

## 3. Results

### 3.1. Participant Characteristics

Maternal and infant characteristics for the 22 breastfeeding dyads in our study are shown in Table 1. All infants were exclusively breastfed at the time of the study. Out of the 22 women, 12 measured their infant’s milk intake at 3 months, 3 at 4 months, 6 at 5 months, and 1 woman at 6 months of lactation. The number of breastfeeds/24 h (breastfeed = feed from one breast) and infant milk intake were within the reported typical ranges for breastfeeding infants 1–6 months of age [21]. A total of 505 milk samples were received and analysed.

### 3.2. Within-Feed Variation

Pre- and post-feed concentrations of leptin, adiponectin, insulin, fat, and glucose are shown in Table 2. Compared to post-feed samples, pre-feed samples had significantly higher concentrations of adiponectin and glucose and lower concentrations of insulin and fat (Table 2).

### 3.3. Circadian Variation in Human Milk Feed Volumes

**Figure 1:**
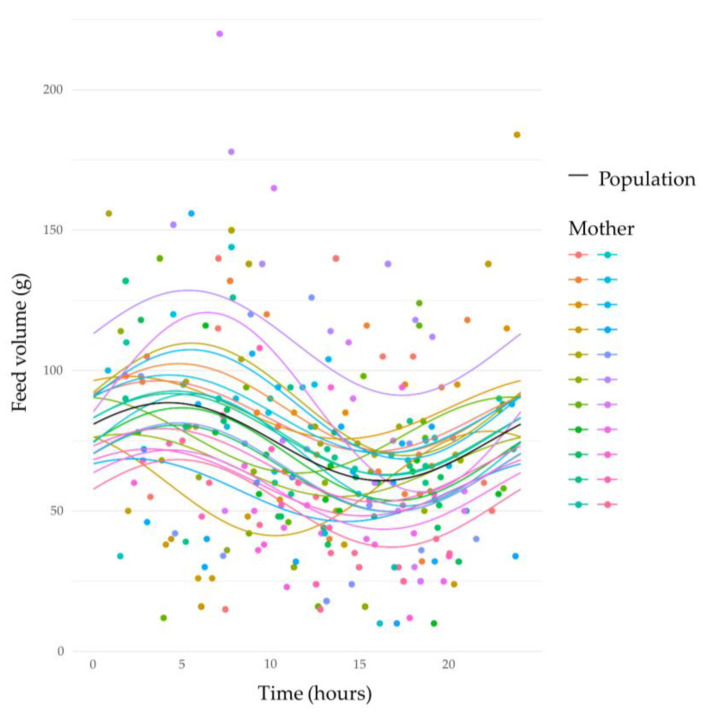
Changes in human milk feed volume (g) over a 24 h period. Coloured dots represent individual mother’s data with the corresponding coloured best-fit line; solid black line indicates the population average.

HM feed volumes exhibited a marked 24 h circadian rhythm (sine, *p* < 0.001; cosine, *p* < 0.04). Feed volumes decreased across the day between 10:00 and 16:00 h, and then increased, peaking in the early morning between 3:00 and 6:00 h (Figure 1).

### 3.4. Circadian Variation in Human Milk Component Concentrations

**Figure 2:**
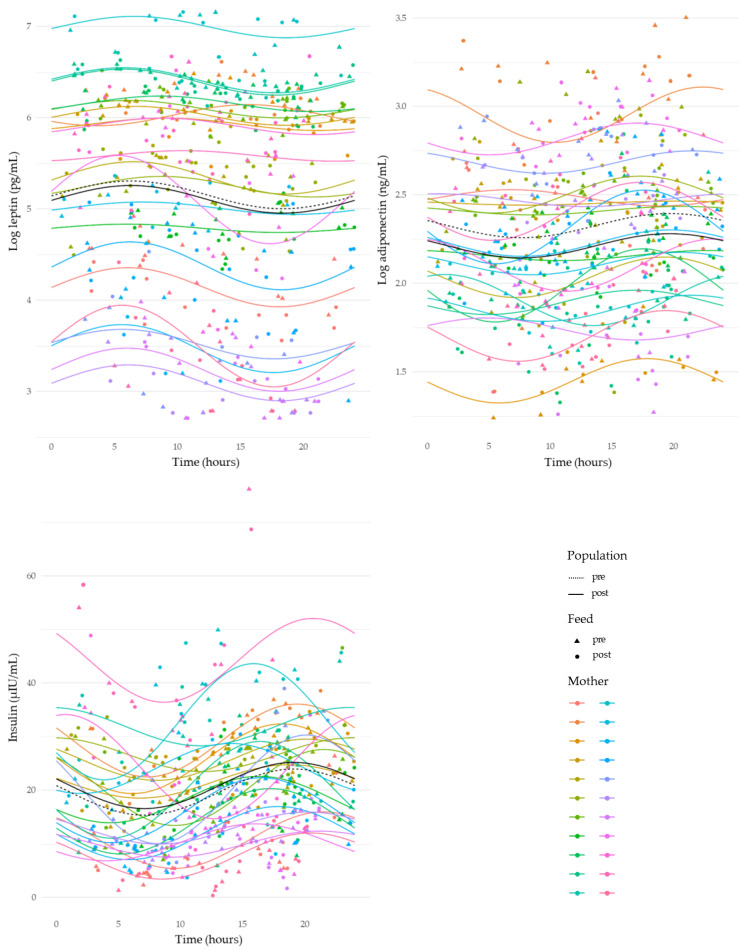
Changes in concentrations of human milk leptin, adiponectin, and insulin over a 24 h period. Average pre-feed sample concentration circadian rhythm is indicated by black dashed line. Average post-feed sample concentration circadian rhythm is indicated by black solid line. Individual mother’s concentrations and rhythms indicated by coloured dots and lines, respectively.

**Figure 3:**
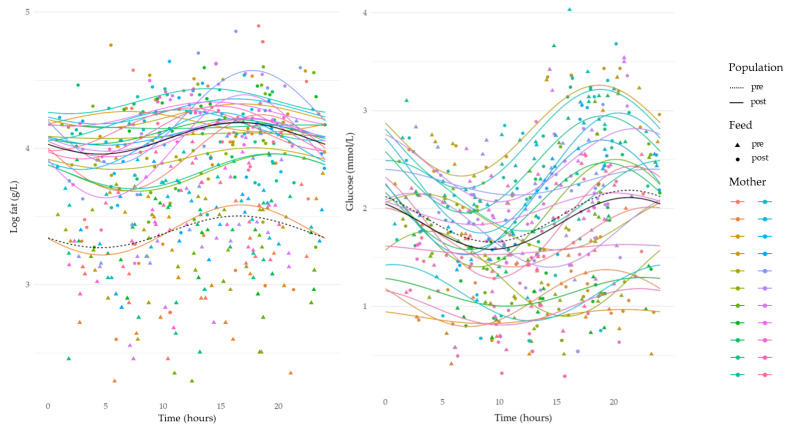
Changes in concentrations of human milk fat and glucose over a 24 h period. Average pre-feed sample concentration circadian rhythm is indicated by black dashed line. Average post-feed sample concentration circadian rhythm is indicated by black solid line. Individual mother’s concentrations and rhythms indicated by coloured dots and lines, respectively.

HM leptin revealed an overall variation across a 24 h period (sine, *p* < 0.001). Leptin concentrations decreased from 12:00 to 17:00 h, and then increased between 20:00 and 06:00 h, with a peak around 05:00 h (Figure 2). Adiponectin, insulin, and glucose concentrations demonstrated strong circadian rhythms, where concentrations increased during the day from 10:00 to 20:00 h, and then decreased from late evening ~ 22:00 h until morning ~07:00 h (adiponectin: sine, *p* = 0.01; insulin: sine, *p* < 0.001; and glucose: sine, *p* = 0.008 and cosine, *p* < 0.01) (Figure 2 and Figure 3). Fat concentration exhibited a marked circadian rhythm increasing throughout the day from 10:00 h to 20:00 h and decreasing throughout the night from 20:00 h to 08:00 h (sine, *p =* 0.002) (Figure 3).

### 3.5. Circadian Variation in Intakes of Human Milk Components

**Figure 4:**
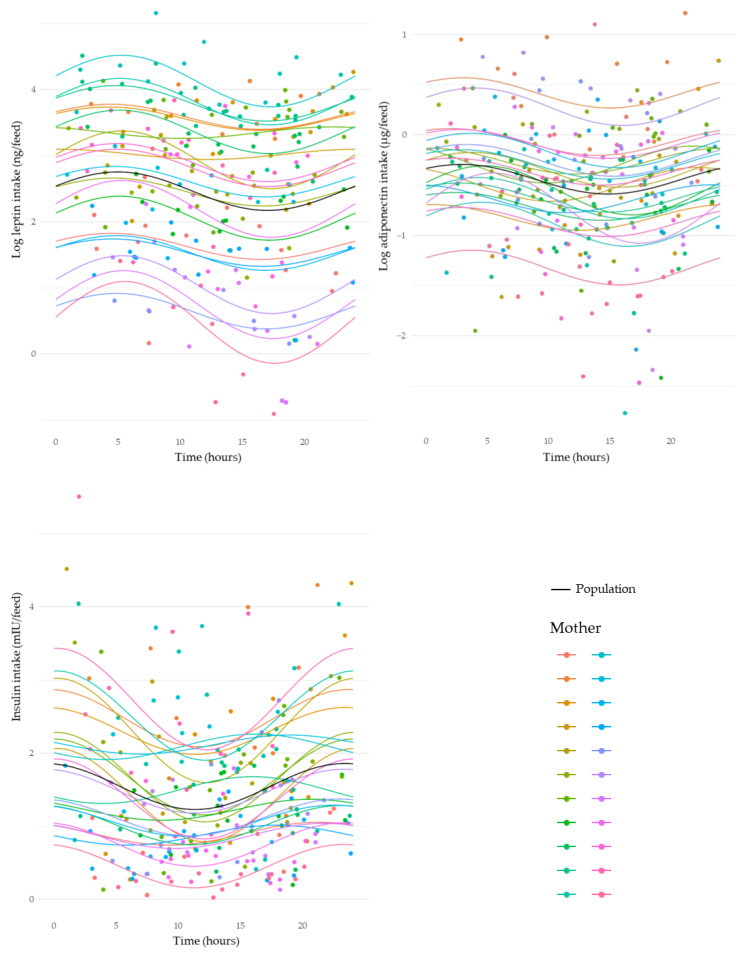
Changes in intakes of human milk leptin, adiponectin, and insulin over a 24 h period. Average circadian rhythm is indicated by black lines. Individual component intakes and rhythms indicated by coloured dots and lines, respectively.

**Figure 5:**
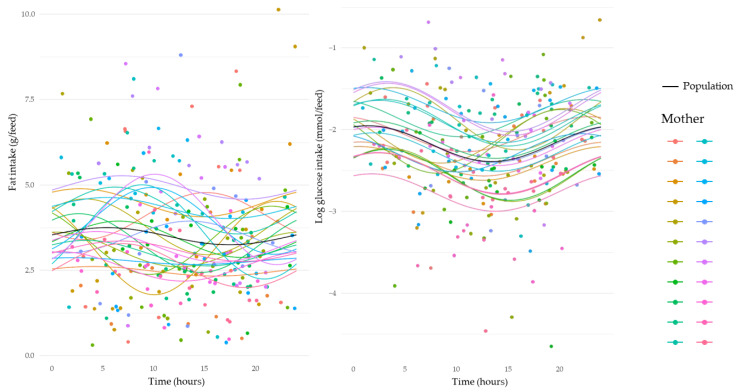
Changes in intakes of human milk fat and glucose over a 24 h period. Average circadian rhythm is indicated by black lines. Individual component intakes and rhythms indicated by coloured dots and lines, respectively.

HM leptin, insulin, and glucose intakes revealed significant circadian rhythms similar to their concentrations across a 24 h period. Leptin intakes decreased from 08:00 h to 16:00 h, and then increased between 20:00 h and 06:00 h, with an evident peak around 05:00 h (sine, *p* < 0.001) (Figure 4 and Figure 5). Insulin intakes increased from ~13:00 h and decreased again after midnight (cosine, *p* = 0.006) (Figure 4). Glucose intakes started to decrease from ~05:00 h and then increased after 13:00 h (cosine, *p* < 0.001) (Figure 5). Adiponectin intakes also exhibited circadian variation, with a clear decrease after ~05:00 h and increase after 15:00 h (sine, *p* = 0.04) (Figure 4).

## 4. Discussion

This study shows, for the first time, that concentrations and infant intakes of HM leptin, insulin, and glucose display circadian rhythms. The circadian rhythm of adiponectin intake is opposite to the circadian rhythm of adiponectin concentration. While HM fat concentration has an evident circadian rhythm, no circadian rhythm was established for fat intake. Additionally, the study found significant differences in pre- and post-feed concentrations of adiponectin, insulin, glucose, and fat. These findings will inform the development of HM sampling guidelines with respect to the timing of sample collection. Further, knowledge of circadian variations in concentrations and intakes of HM leptin, adiponectin, insulin, glucose, and fat will enable further investigation of HM as chrono-nutrition, and its impact on infant health and development.

The variation in HM fat concentration is well documented; however, the majority of studies reporting circadian rhythm of fat concentration compared concentrations at several time points rather than for all breastfeeds over a 24 h period. Our study is the first to investigate circadian variation in fat concentration using 24 h data and has found that HM fat concentration peaks around 17:00 h (Figure 3). This result is in agreement with the literature indicating that evening HM samples have a higher fat concentration [11,21,24], likely due to the breast being more drained of milk at that time. Regardless of the circadian rhythm of fat concentration, HM fat intakes remained constant despite variations in feed volume (Figure 5). Since fat is the prime source of HM energy, infants might control their appetite by controlling their feed volumes and their fat intake [25,26]. The circadian variation in HM fat concentration could also be explained by changes in the volume of milk available in the breast before and after each feed [27]. Specifically, when there is a particular circadian pattern of milk removal, this pattern may result in a specific circadian pattern of fat concentration [27].

HM insulin is derived from the maternal circulation [28] and was found to have a higher concentration in post-feed samples peaking in the early evening (Figure 2). Similarly, we found the insulin intake to display circadian variations that increased after 12:00 h and reached a peak around midnight (Figure 4). Thus, the circadian rhythms of HM insulin concentration and intake reported here likely reflect maternal serum insulin concentration. This is congruent with previous observations that the maternal physiological environment plays a crucial role in the regulation of HM hormone composition [28,29]. A direct comparison of the circadian rhythm of HM and maternal serum insulin concentrations is warranted.

This study’s findings for glucose are also in agreement with those of a previous study showing the lowest HM concentrations occurring between 10:00 h and 14:00 h [30]. We found that a nadir of HM glucose concentration occurs around 10:00 h (Figure 3), while the HM glucose intake nadir is between 10:00 and 15:00 h (Figure 5). These results contrast with those of Lammi-Keefe et al. [31], who found no change in glucose concentration throughout the day; however, this is likely due to the limited sample size (*n* = 6). Direct comparison of HM and maternal plasma glucose suggests that increases in HM glucose concentration commence 40 to 90 min after an increase in maternal plasma glucose concentration, returning to baseline 120 to 150 min after maternal return of baseline concentration [32]. Therefore, HM glucose concentrations appear to have a delayed response to maternal plasma glucose concentration rhythms since it starts to rise from 10:00 h. In adults, glucose concentration is relatively stable between 01:00 and 05:30 h and increases during the morning [33,34]. Future studies should include information on maternal meal/liquid ingestion to clarify the circadian pattern shown in this study.

Circadian rhythms in skim HM leptin concentration have been shown to be significantly higher between 22:00 and 04:00 h [35], which is consistent with the nocturnal rise in human circulating leptin [36,37,38,39,40]. Indeed, we confirm a nocturnal rise in both whole milk leptin concentration and intake (Figure 2 and Figure 4), and no significant concentration differences between pre- and post-feed samples over the course of 24 h. Further, this nocturnal rise supports previous reports that HM leptin is derived largely from maternal circulation [41,42]. In addition, we observed a matched nocturnal increase in HM leptin intake. However, HM leptin intake reflects what is received by the infant and measuring the intake will contribute to more comprehensive investigations of the impact of HM leptin on infant outcomes.

For the first time, we found that HM adiponectin concentration has a nocturnal decline that starts in the late evening and reaches a nadir in the early morning (Figure 2). This circadian pattern is similar to that previously reported for serum adiponectin concentrations in healthy men [43]. When comparing circadian rhythms of HM adiponectin intake and concentration, it is apparent that HM adiponectin intake has a circadian pattern peaking in the early morning and then declining to its lowest point in the early afternoon (Figure 4). Similarly, evidence of adiponectin coding gene (*ADIPOQ*) expression in subcutaneous adipose tissue suggests that the adiponectin circadian rhythm has a nocturnal rise with a peak in the early morning and a nadir between afternoon and early evening [44]. Indeed, adiponectin has previously been significantly correlated with HM fat concentration [45]; the nocturnal rise in HM adiponectin intake could be explained by the effect of HM feed volume and associated fat concentration circadian rhythms since adiponectin is mainly secreted by adipose tissue.

Interestingly, commercial milk formula has constant component concentrations, while HM components have variable concentrations and intakes over time. Studies comparing HM with commercial milk formula found that the latter does not contain leptin, insulin, testosterone, thyroid-stimulating hormone (TSH) or thyroxine [46,47,48]. The role of circadian rhythms of HM leptin, insulin, adiponectin, glucose, and fat is not known. However, they may act as programming signals for infant circadian rhythms and breastfed infants might gain benefits by coordinating with the maternal circadian rhythm [49]. Further exploration is needed for understanding the impact of the HM circadian rhythm as signals of metabolic programming and recognition of the differences between circadian rhythms of breastfed infants and those fed commercial milk formula.

In our comparison of the circadian rhythm of HM component concentrations and intakes, using the actual intake volume of each feed, we provide important data for the re-evaluation of current HM sampling protocols. Knowledge of the effects of sampling time on concentrations of HM components is lacking in the literature. Collecting HM in the morning is a common sampling procedure, which can also include maternal overnight fasting [19,20]. Therefore, it could be assumed that sampling at one time-point is potentially not sufficient when investigating HM components in relation to maternal factors and their influence on infant outcomes.

A considerable strength of this study is the collection of both pre- and post-feed samples and breastfeed volumes measured across a 24 h time period, to enable the calculation of infant intake. This study design permitted us to assess both within-feed and throughout-the-day variations. In addition, all women were exclusively breastfeeding. A limitation of this study is that no recording of maternal nutritional intake was conducted along with sample collection. Overall, our primary objective was to investigate the within-feed variation in HM components and the presence or absence of circadian variation in HM composition. Therefore, the study design is relevant to the objectives of the study. Investigation of the factors that control HM circadian rhythms will require concurrent maternal plasma sampling, the recording of maternal diet, and milk sample collection.

## 5. Conclusions

Our findings indicate that HM leptin, adiponectin, insulin, fat, and glucose concentrations display circadian rhythms. Furthermore, intakes of these components, except for fat, also demonstrate circadian fluctuations. The sampling of HM is complicated by circadian rhythms and differences in pre- and post-feed concentrations of insulin, adiponectin, fat, and glucose. These findings are of significant value for the design of future studies that aim to investigate the role of HM as chrono-nutrition.

## Figures and Tables

**Table 1:** Participant characteristics.

Characteristics	*n* = 22
Maternal	
Age (years)	32.7 ± 5.5 ^a^ (25.0–46.0)
BMI (kg/m^2^)	27.1 ± 6.0 (19.2–38.8)
Infant	
Sex (male, female)	12, 10
Gestational age (weeks)	38.9 ± 1.4 (36.0–41.0)
Birth weight (g)	3453 ± 398 (4455–2940)
Breastfeeding characteristics	
Time postpartum (months)	3.8 ± 1.0 (3.0–6.0)
24 h milk intake (g)	879 ± 309 (455–1850)
Number of breastfeeds per 24 h	12.1 ± 3.5 (7.0–16.0)

^a^ Data are mean ± standard deviation (SD) and minimum–maximum.

**Table 2:** Pre-and post-feed concentrations of human milk components.

Components	Pre-Feed, (*n*)	Post-Feed, (*n*)	Estimate ± SE	*p*-Value ^c^
Log leptin, pg/mL	2.28 ± 0.52 ^a^, (234)	2.27 ± 0.50, (232)	0.05 ± 0.03 ^b^	0.16
Log adiponectin, ng/mL	1.01 ± 0.19, (251)	0.96 ± 0.20, (248)	0.11 ± 0.02	<0.001
Insulin, µIU/mL	19.45 ± 11.00, (236)	20.83 ± 11.30, (234)	−1.24 ± 0.54	0.02
Log fat, g/L	1.47 ± 0.18, (241)	1.77 ± 0.16, (240)	−0.69 ± 0.03	<0.001
Glucose mmol/L	1.91 ± 0.73, (247)	1.84 ± 0.72, (243)	0.07± 0.03	0.02

Data are ^a^ mean ± SD and ^b^ parameter estimate and standard error of measurement. ^c^ *p*-values are from linear mixed effects models.

## Data Availability

Data sharing not applicable.
